# Impact of physical activity on surrogate markers of cardiovascular disease in the haemodialysis population

**DOI:** 10.1093/ckj/sfae198

**Published:** 2024-06-28

**Authors:** Katherine L Hull, Lucy Abell, Sherna F Adenwalla, Roseanne E Billany, Stephanie Burns, James O Burton, Darren Churchward, Matthew P M Graham-Brown, Laura J Gray, Patrick Highton, Courtney J Lightfoot, Rahma Said, Alice C Smith, Hannah M L Young, Daniel S March

**Affiliations:** Department of Cardiovascular Sciences, University of Leicester, Leicester, UK; John Walls Renal Unit, University Hospitals of Leicester NHS Trust, Leicester, UK; Department of Population Health Sciences, University of Leicester, Leicester, UK; Department of Cardiovascular Sciences, University of Leicester, Leicester, UK; John Walls Renal Unit, University Hospitals of Leicester NHS Trust, Leicester, UK; Department of Cardiovascular Sciences, University of Leicester, Leicester, UK; University Hospitals of Leicester NHS Trust, Leicester, UK; Department of Cardiovascular Sciences, University of Leicester, Leicester, UK; John Walls Renal Unit, University Hospitals of Leicester NHS Trust, Leicester, UK; School of Sport, Exercise and Health Sciences, Loughborough University, Loughborough, UK; Department of Cardiovascular Sciences, University of Leicester, Leicester, UK; Department of Cardiovascular Sciences, University of Leicester, Leicester, UK; John Walls Renal Unit, University Hospitals of Leicester NHS Trust, Leicester, UK; School of Sport, Exercise and Health Sciences, Loughborough University, Loughborough, UK; Department of Population Health Sciences, University of Leicester, Leicester, UK; NIHR Biomedical Research Centre, Leicester, UK; Diabetes Research Centre, Leicester General Hospital, University of Leicester, UK; NIHR Biomedical Research Centre, Leicester, UK; Leicester Kidney Lifestyle Team, Department of Population Health Sciences, University of Leicester, UK; Department of Population Health Sciences, University of Leicester, Leicester, UK; NIHR Biomedical Research Centre, Leicester, UK; Leicester Kidney Lifestyle Team, Department of Population Health Sciences, University of Leicester, UK; University Hospitals of Leicester NHS Trust, Leicester, UK; NIHR Biomedical Research Centre, Leicester, UK; Diabetes Research Centre, Leicester General Hospital, University of Leicester, UK; Department of Cardiovascular Sciences, University of Leicester, Leicester, UK

**Keywords:** cardiac magnetic resonance imaging, cardiovascular disease risk, exercise, haemodialysis, physical activity

## Abstract

**Background:**

The haemodialysis (HD) population is sedentary, with substantial cardiovascular disease risk. In the general population, small increases in daily step count associate with significant reductions in cardiovascular mortality. This study explores the relationship between daily step count and surrogate markers of cardiovascular disease, including left ventricular ejection fraction (LVEF) and native T1 (a marker of diffuse myocardial fibrosis), within the HD population.

**Methods:**

This was a post hoc analysis of the association between daily step count and metabolic equivalent of task (MET) and prognostically important cardiac magnetic resonance imaging parameters from the CYCLE-HD study (ISRCTN11299707). Unadjusted linear regression and multiple linear regression adjusted for age, body mass index, dialysis vintage, haemoglobin, hypertension and ultrafiltration volume were performed. Significant relationships were explored with natural cubic spline models with four degrees of freedom (five knots).

**Results:**

A total of 107 participants were included [age 56.3 ± 14.1 years, 79 (73.8%) males]. The median daily step count was 2558 (interquartile range 1054–4352). There were significant associations between steps and LVEF (β = 0.292; *P* = .009) and steps and native T1 (β = −0.245; *P* = .035). Further modelling demonstrated most of the increase in LVEF occurred at up to 2000 steps/day and there was an inverse dose–response relationship between steps and native T1, with the most pronounced reduction in native T1 between ≈2500 and 6000 steps/day.

**Conclusions:**

The results suggest an association between daily step count and parameters of cardiovascular health in the HD population. These findings support the recommendations for encouraging physical activity but are not the justification. Further research should evaluate whether a simple physical activity intervention improves cardiovascular outcomes in individuals receiving maintenance HD.

KEY LEARNING POINTS **What was known:**

- Cardiovascular disease is the leading cause of mortality in the prevalent haemodialysis (HD) population.
- Individuals receiving maintenance HD are sedentary compared with both the general population and earlier stages of chronic kidney disease.
- Small increases in daily physical activity have been associated with significant reductions in all-cause and cardiovascular mortality in the general population.

**This study adds:**

- Higher daily step count associated with an increase in left ventricular ejection fraction (LVEF) and reduced global native T1 time (a marker of diffuse myocardial fibrosis) on cardiac magnetic resonance imaging, two prognostically important cardiovascular disease markers.
- The number of steps associated with improvements in LVEF and global native T1 time were modest: a marked increase in LVEF was associated with 0–2000 steps/day and the most pronounced reduction in native T1 was associated with 2500–6000 steps/day.

**Potential impact:**

- The findings suggest that small improvements in daily physical activity levels potentially have a large impact on cardiovascular health, but further work through longitudinal studies and randomized clinical trials are needed to confirm the associations.
- For many, increasing the daily step count is an accessible, cost-effective and easy way to enhance daily physical activity levels.
- Clinicians should be confident in advising increasing physical activity levels in the HD population, with the knowledge that those starting from a low-level may stand to benefit the most.

## INTRODUCTION

Individuals with end-stage kidney disease (ESKD) receiving haemodialysis (HD) have a significantly increased risk of cardiovascular disease (CVD) [1], which is the leading cause of death in this population [2]. Strategies to mitigate CVD risk have little impact, as pathophysiological processes are driven by non-traditional CVD risk factors such as chronic inflammation, uraemia, anaemia, renal bone disease and HD-induced myocardial stunning [3–5]. Subsequently, the myocardium undergoes remodelling, resulting in cardiomyopathy, impaired left ventricular function and myocardial fibrosis [3].

Individuals receiving maintenance HD are physically inactive compared with both the general population and those with earlier stages of chronic kidney disease (CKD) [6, 7]. Low levels of physical activity strongly associate with CVD and mortality in this population [8–10]. However, the precise relationship between physical activity and CVD remains unclear. In the general population without known CVD, regular physical activity has been associated with the preservation of surrogate markers for cardiovascular health on cardiac magnetic resonance imaging (CMRI), e.g. left ventricular ejection fraction (LVEF), compared with those who are physically inactive [11]. Furthermore, small increases in daily physical activity with a daily step count ≥2300/day associates with reductions in all-cause and CVD mortality in the general population [12]. Currently it is unclear if higher levels of physical activity associate with improved measures of cardiovascular health in the HD population.

In line with the UK Chief Medical Officers guideline [13], the UK Kidney Association (UKKA) clinical practice guideline of exercise and lifestyle in CKD recommends that individuals receiving HD should aim for 150 minutes of moderate-intensity activity per week (or 75 minutes of vigorous activity) [13]. These recommendations are based largely on cohort data from individuals who have self-reported physical activity levels [8, 10, 14], so despite being graded as a strong recommendation, they are of low-quality evidence [13]. It has previously been suggested that 3000–4000 steps/day reliably approximates 30 minutes of continuous moderate-intensity walking (as recommended by the guidelines) [15] and that individuals living with chronic disease should aim for at least 5000 steps/day in total [16], as this level of activity has been proposed as the threshold for a ‘sedentary lifestyle index’. Further understanding of the level of physical activity that associates with improved cardiovascular health in the ESKD population is required.

This study investigated the relationship between levels of physical activity, assessed by average steps per day and metabolic equivalent of task (MET), and CMRI measures of CVD in individuals receiving maintenance in-centre HD.

## MATERIALS AND METHODS

This is a post hoc analysis of baseline data from participants enrolled in the CYCLE-HD trial (ISRCTN11299707; Improving cardiovascular health in dialysis patients using a structured programme of exercise) [17]. Detailed descriptions of the trial design, including specific inclusion and exclusion criteria and data collection procedures, are described elsewhere [18]. Briefly, individuals ≥18 years of age receiving maintenance HD for ≥3 months were eligible for inclusion. Major exclusion criteria included inability to participate in the current exercise program due to perceived physical or psychological barriers, unable to undergo CMRI and unfit to undertake exercise according to the American College of Sports Medicine guidelines [19]. As part of their baseline assessment, participants were comprehensively phenotyped, including with CMRI (the gold standard for assessment of cardiovascular structure and function) and objectively derived physical activity from accelerometers. The trial was given ethical approval by the National Health Service (NHS) Research Ethics Committee East Midlands (Northampton, UK; REC ref: 14/EM/1190). The trial was conducted according to the Declaration of Helsinki and all participants provided written informed consent.

### Sample size

The CYCLE-HD trial recruited 155 maintenance HD patients [17, 18]. No *a priori* sample size calculation was completed for this post hoc analysis.

### Physical activity

Levels of physical activity were measured using the SenseWear Pro3 Armband (BodyMedia, Pittsburgh, PA, USA), an accelerometer with physiological sensors. Participants were instructed to wear the accelerometer for 7 days on the upper arm (the opposite arm of their vascular access). They were instructed to remove the equipment only for bathing or any other water activity. Accelerometer data were included for analysis as long as it was worn for a minimum of 3 days and included at least one HD day and two non-HD days as previously recommended [20]. The total steps taken and average METs were calculated. For each participant, steps per day data were calculated as the average for total days worn (i.e. the total number of steps divided by days worn).

### Cardiovascular outcome assessment

Participants underwent CMRI as part of the CYCLE-HD trial on a Skyra 3T platform (Siemens Medical Imaging, Erlangen, Germany). Scans were performed on non-dialysis days 18–24 hours after the most recent dialysis treatment, avoiding the long interdialytic gap, to standardise fluid status. Scans were anonymised before being randomly allocated for off-line analysis by a single, blinded assessor. LVEF, LV mass, LV mass index (LVMI), LV mass/LV end diastolic volume (LVM/LVEDV), aortic pulse wave velocity (PWV) and native T1 mapping were acquired using CMRI, with acquisition and analysis performed in accordance with international recommendations [21]. Full details of the acquisition sequences and analysis of CMRI parameters have been published previously [18].

### Data collection time frame

Baseline data only (completed prior to randomisation) were used for this post hoc analysis. Follow-up data were not used due to the impact of the intervention (intradialytic cycling) on the CMRI surrogate markers of CVD.

### Statistical analysis

All data are presented as mean [± standard deviation (SD)] or median [interquartile range (IQR)]. Data were checked for normality by observing the histogram plot and the *P*-value from the Shapiro–Wilk test. Unadjusted linear regression of the physical activity and CMRI parameters were performed. Significant associations were further explored using a multiple linear regression, adjusting for age, body mass index (BMI), hypertension, haemoglobin level, dialysis vintage and ultrafiltration volume (i.e. dialysis-specific non-traditional cardiovascular risk factors). To further investigate associations between CMRI and physical activity parameters, the relationships were modelled using natural cubic splines with a range of degrees of freedom (2–7) considered. Spline modelling was completed using RStudio (Posit Software, Boston, MA, USA). All other analyses were performed using SPSS for Windows version 28.0 (IBM, Armonk, NY, USA). Statistical significance was accepted at *P* < .05.

## RESULTS

The CYCLE-HD trial enrolled 155 participants from March 2015 through April 2018; 130 participants completed the baseline assessments and were randomised to either the intervention or control (*n* = 65 in each group). There were 108 participants with physical activity data (average steps per day and METS) and CMRI parameters. One participant completed an average of 19 905 steps/day, which was +2.5 SD away from the group mean. This participant was removed from the analysis.

The demographics of the remaining 107 participants included in this post hoc analysis are presented in Table 1. The median daily step count was 2558 (IQR 1054–4352) and the median METs was 1.2 (IQR 1.0–1.4).

**Table 1:** Demographics of CYCLE-HD participants (*N* = 107) at baseline with CMRI and average daily steps data.

Variable	Values
Age (years), mean ± SD	56.3 ± 14.1
Male, *n* (%)	79 (73.8)
Dialysis vintage (years), median (IQR)	1.1 (0.4–3.4)
Interdialytic SBP (mmHg), mean ± SD	146 ± 36
Interdialytic DBP (mmHg), mean ± SD	69 ± 16
Pre-dialysis SBP (mmHg), mean ± SD	139 ± 22
Pre-dialysis DBP (mmHg), mean ± SD	73 ± 12
Haemoglobin (g/l), mean ± SD	113 ± 13
Parathyroid hormone (pmol/l), median (IQR)	29.2 (14.4–77)
Diabetes mellitus diagnosis, *n* (%)	39 (36.4)
CVD^a^, *n* (%)	77 (72.0)
Smoker (current or previous), *n* (%)	54 (50.5)
BMI (kg/m^2^), median (IQR)	26.3 (23.0–29.8)
Ultrafiltration volume (ml), median (IQR)	2350 (1600–2800)
Daily step count, median (IQR)	2558 (1054–4352)
METs, median (IQR)	1.2 (1.0–1.4)
LVEF (%),median (IQR)	55.9 (47.7–61.0)

SBP: systolic blood pressure; DBP: diastolic blood pressure.

^a^History of CVD refers to one or more of peripheral vascular disease, atrial fibrillation, myocardial infarction, ischaemic heart disease, heart failure, stroke or trans-ischaemic attack and hypertension.

### Associations between steps per day and CMRI parameters

There was a significant positive association between baseline step count and LVEF (β = 0.247; *P* = .010). There was a significant negative association between baseline step count and native T1 (β = −0.272; *P* = .006). There were no significant associations between other CMRI variables and step count (Table 2).

**Table 2:** Unadjusted linear regression between CMRI parameters of physical activity: average daily step counts and METs.

	Baseline steps		Baseline METs	
CMRI parameter	β	*P*-value	β	*P*-value
LVEF (%)	0.247	.010*	0.109	.263
LVM (g)	−0.031	.754	0.119	.222
LVEDV (g/ml)	−0.043	.658	−0.024	.804
Global native T1 (msec)	−0.272	.006*	−0.213	.032*
PWV (per msec)	−0.169	.098	−0.288	.004*

^a^Denotes significant associations.

**Figure 1:**
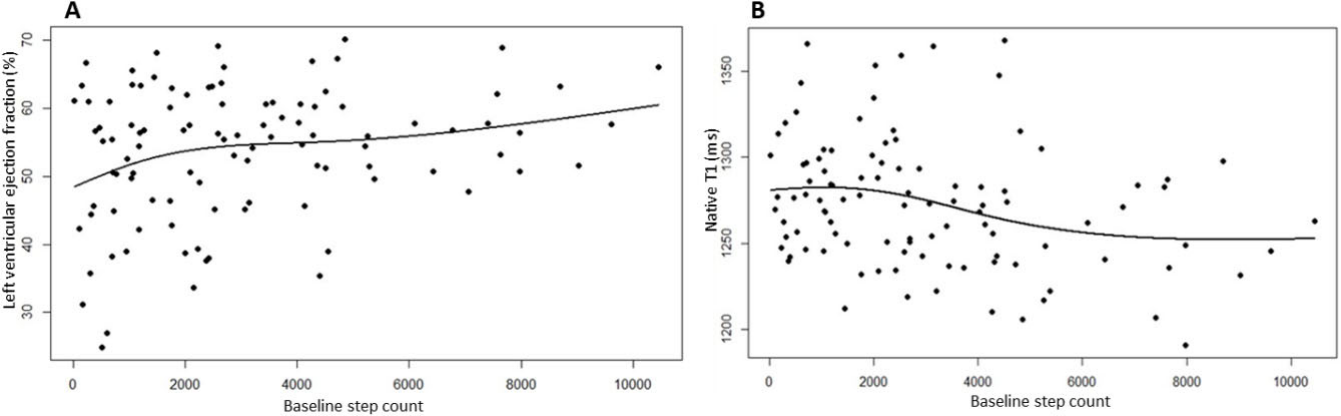
(**A**) Modelling of baseline step count against LVEF. The increase in LVEF occurs at 0–2000 steps. (**B**) Modelling of baseline step count against global native T1. The decrease in native T1 time occurs at 2500–5000 steps.

After adjustment for covariates (age, BMI, sex, history of CVD, haemoglobin levels, dialysis vintage and ultrafiltration volume), the positive association between baseline steps and LVEF (β = 0.292; *P* = .009) persisted (Table 3). Further modelling of this relationship using a natural cubic spline model with five knots (three internal and two boundary knots) showed that most of the increase in LVEF occurred between 0 and 2000 steps/day (Fig. 1A). Four and five knots produced similar results, giving a relationship beyond a linear one but not appearing to overfit the data. The negative association between baseline steps and native T1 (β = −0.245; *P* = .035) also persisted (Table 4). Natural cubic spline models showed an inverse dose–response association with the most pronounced reduction in native T1, with the sharpest phase of the curve between ≈2500 and 6000 steps/day (Fig. 1B).

**Table 3:** Association between LVEF and baseline step count, adjusted for covariates (age, BMI, sex, history of CVD, haemoglobin level, dialysis vintage and ultrafiltration volume).

Model summary								
*R*	*R* ^2^	Adjusted *R*^2^	SE	*R* ^2^ change	ANOVA *F*	df1	df2	*P*-value
0.419	0.176	0.108	9.204	0.176	2.587	8	97	0.013*
Independent variable			β			*P*-value		
Baseline steps			0.292			.009*		
Age			0.058			.603		
BMI			0.115			.227		
Sex			0.297			.003*		
CVD			0.006			.952		
Haemoglobin level			0.025			.798		
Dialysis vintage			0.053			.582		
Ultrafiltration volume			−0.016			.873		

ANOVA: analysis of variance; df: degrees of freedom; SE: standard error.

*Denotes significant associations.

**Table 4:** Association between global native T1 and baseline step count, adjusted for covariates (age, BMI, sex, history of CVD, haemoglobin levels, dialysis vintage and ultrafiltration volume).

Model summary								
*R*	*R* ^2^	Adjusted *R*^2^	SE	*R* ^2^ change	ANOVA F	df1	df2	*P*-value
0.391	0.153	0.079	36.046	0.153	2.069	8	92	0.047*
Independent variable	β	*P*-value						
Baseline steps	−0.245	.035*						
Age	0.088	.449						
BMI	−0.250	.013*						
Sex	−0.022	.832						
CVD	−0.095	.338						
Haemoglobin level	−0.057	.568						
Dialysis vintage	−0.015	.878						
Ultrafiltration volume	0.016	.874						

ANOVA: analysis of variance; df: degrees of freedom; SE: standard error.

*Denotes significant associations.

### Association between METs and CMRI parameters

There were significant negative associations between METs and native T1 (β = −0.213; *P* = .032) and PWV (β = −0.288; *P* = −0.004). The remaining CMRI markers did not have a significant association with baseline METs (Table 2).

On adjustment for covariates (age, BMI, sex, history of CVD, haemoglobin levels, dialysis vintage and ultrafiltration volume), the association between METs and native T1 (β = −0.170; *P* = .187) and PWV (β = −0.031, *P* = .786) were no longer significant.

## DISCUSSION

This study demonstrates that individuals receiving in-centre maintenance HD are inactive, with a low average daily step count and METs. Higher daily step count is associated with an increase in LVEF and reduced global native T1 time (a marker of diffuse myocardial fibrosis [22, 23]); this association remained while controlling for some non-traditional CVD risk factors (dialysis vintage and ultrafiltration volume) unique to the HD population. Further modelling of the data suggested a marked increase in LVEF was associated with 0–2000 steps/day and the most pronounced reduction in native T1 was associated with 2500–6000 steps/day.

The findings from this study corroborate the physical activity levels previously reported for chronic disease and HD populations. There is a clear relationship between low physical activity levels and increased risk of CVD [24]. Chronic non-communicable diseases are often characterised by physical inactivity, promoting deconditioning and loss of functional capacity [25]. This downward spiral of physical inactivity and endemic sedentary behaviour is well recognised in the HD population [7], with physical activity levels well below the healthy sedentary population [6] and associated with both cardiovascular and all-cause mortality [8, 9, 26].

The UKKA guideline takes into consideration the location of activity (i.e. intradialytic versus interdialytic activity) and suggests that some physical activity is better than none, however, it provides neither guidance nor suggestions on the type of activity and advises professional supervision while increasing activity levels [13]. Emerging evidence questions aspects of these recommendations and undermines the definition of the sedentary behaviour index [16]. A meta-analysis of >225 000 participants from six countries demonstrated an increment in daily step count of 500 reduced the risk of cardiovascular mortality by 7% and cardiovascular health benefits were observed from a daily step count ≥2500 [12]. Similarly, in a large cohort study of >70 000 participants from the UK biobank, a daily step count ≥2200 was associated with lower mortality and CVD risk [27]. The results of this exploratory study support these findings within a prevalent HD cohort: small increases in steps per day within the individual's usual and (most probably) unsupervised/personal time is associated with improved surrogates of CVD.

Increased physical activity levels via low-impact aerobic exercise, such as walking, improves traditional CVD risk factors, including lipid levels, insulin sensitivity, blood pressure and weight management [28]. Strategies to mitigate traditional CVD risk factors do not improve cardiovascular outcomes in the HD population as they do in the general population [29, 30]. Non-traditional risk factors, such as chronic inflammation and HD-induced myocardial stunning, are major contributors to pathological myocardial remodelling [5, 31, 32]. Performing physical activity through planned exercise may mediate reductions in some non-traditional risk factors through a number of mechanisms, including improved intradialytic cardiovascular stability [33, 34], release of cardioprotective myokines from skeletal muscle [35], reductions in oxidative stress, shifts to less inflammatory cell and cytokine profiles and reduced recruitment of monocytes into adipose tissue [36]. Thus it is biologically plausible that individuals receiving maintenance HD with higher physical activity levels have less evidence of structural CVD. This is corroborated by the findings of the main CYCLE-HD trial where intradialytic exercise significantly reduced LVM and native T1 mapping [17].

There was not a significant association between participants’ METs and the cardiovascular surrogate markers on CMRI, LVEF and native T1 mapping. The median METs for the cohort was 1.2 (IQR 1.0–1.4). This low METs alongside the narrow variance reflects the majority of participants’ physical activity levels were of low intensity. In contrast, there was greater variance in the volume of physical activity, as reflected by the median daily step count of 2558 (IQR 1054–4352). This may partly explain the lack of association between intensity of the physical activity (as represented by METs) and the CMRI parameters.

There are a number of limitations to this study that need to be considered when interpreting the findings. This study is an exploratory post hoc analysis; it was not a pre-specified assessment of the original CYCLE-HD data. However, the data were collected prospectively from multiple dialysis units. There is a lack of granularity in the steps data; it is unknown how, when and at what intensity the steps were completed, there is little insight into the physical activity behaviour patterns (e.g. planned exercise versus habitual physical activity, sitting and standing durations etc.) and it is not possible to account for unrecorded activity. There are other potential confounding factors which it was not possible to account for, including cardiac biomarkers (e.g. natriuretic peptides) and osteoarticular disorders that may influence daily physical activity levels. The outcome measures considered are surrogate markers for CVD and not clinical endpoints, although they are predictors of cardiovascular events. Importantly, this is a cross-sectional study and direction of causation cannot be inferred. Findings may also be attributable to reverse causality, whereby improved cardiovascular status facilitates increased physical activity levels.

Despite the limitations, these findings have positive implications for clinical practice and further research. Behaviour change interventions that focus on specific health-related behaviours are often unsuccessful [37] and the barriers to exercise within the HD population are multifaceted [38]. The UKKA physical activity guidelines are likely to be a daunting prospect for patients who are not currently active, and despite significant efforts within the renal community to incorporate exercise into usual dialysis care (i.e. intradialytic cycling), implementation into clinical practice is lagging. The number of steps per day that associates with improved measures of CVD was relatively low and far below the recommendation for physical activity in the UKKA guideline. This supports the idea that the most physically inactive have the most to gain from increasing their activity levels and even small increases could have an important effect. Certainly these data support a move away from threshold-based recommendations towards the message that some physical activity is better than none [13]. Further research would involve assessing the impact of a simple physical activity intervention on cardiovascular and mortality endpoints through a randomised controlled trial.

## CONCLUSION

The HD population is sedentary, with an overwhelming burden of CVD. Daily step count associates with important structural and functional measures of CVD; benefit was observed at daily step counts >2000, well below the guideline recommendations for physical activity. Lower daily step count associates with important structural and functional measures of CVD, with most of the benefit observed at low levels of daily steps, well below guideline recommendations. For many patients, increasing daily step count is achievable, cost-effective and potentially accessible. Work is needed to explore these findings in longitudinal and interventional studies and to determine the impact on important patient-centred outcomes such as survival and cardiovascular events. Clinicians can be confident in advising increasing physical activity levels, with the knowledge that those starting from a low-level may stand to benefit the most and, for these individuals, small improvements may have large effects.

## Data Availability

Deidentified individual participant data collected for the study and a data dictionary defining each field in the set will be made available to others upon specific request to the chief investigator and corresponding author, provided all regulatory and data sharing approvals are obtained afterwards.

## References

[1] Foley RN, Parfrey PS, Sarnak MJ. Epidemiology of cardiovascular disease in chronic renal disease. J Am Soc Nephrol 1998;9(12 Suppl):S16–23. 11443763

[2] Saran R, Robinson B, Abbott KC et al. US Renal Data System 2016 annual data report: epidemiology of kidney disease in the United States. Am J Kidney Dis 2017;69:A7–8. 10.1053/j.ajkd.2016.12.004 28236831 PMC6605045

[3] Graham-Brown MP, Patel A, Stensel D et al. Imaging of myocardial fibrosis in patients with end-stage renal disease: current limitations and future possibilities. Biomed Res Int 2017;2017:5453606. 10.1155/2017/5453606 28349062 PMC5352874

[4] Kendrick J, Chonchol MB. Nontraditional risk factors for cardiovascular disease in patients with chronic kidney disease. Nat Clin Pract Nephrol 2008;4:672–81. 10.1038/ncpneph0954 18825155

[5] Burton JO, Jefferies HJ, Selby NM et al. Hemodialysis-induced cardiac injury: determinants and associated outcomes. Clin J Am Soc Nephrol 2009;4:914–20. 10.2215/CJN.03900808 19357245 PMC2676185

[6] Johansen KL, Chertow GM, Ng AV et al. Physical activity levels in patients on hemodialysis and healthy sedentary controls. Kidney Int 2000;57:2564–70. 10.1046/j.1523-1755.2000.00116.x 10844626

[7] Wilkinson TJ, Clarke AL, Nixon DG et al. Prevalence and correlates of physical activity across kidney disease stages: an observational multicentre study. Nephrol Dial Transplant 2021;36:641–9. 10.1093/ndt/gfz235 31725147

[8] Stack AG, Molony DA, Rives T et al. Association of physical activity with mortality in the US dialysis population. Am J Kidney Dis 2005;45:690–701. 10.1053/j.ajkd.2004.12.013 15806472

[9] Martins P, Marques EA, Leal DV et al. Association between physical activity and mortality in end-stage kidney disease: a systematic review of observational studies. BMC Nephrol 2021;22:227. 10.1186/s12882-021-02407-w 34144689 PMC8212466

[10] Johansen KL, Kaysen GA, Dalrymple LS et al. Association of physical activity with survival among ambulatory patients on dialysis: the comprehensive dialysis study. Clin J Am Soc Nephrol 2013;8:248–53. 10.2215/CJN.08560812 23124787 PMC3562868

[11] Schafnitzel A, Lorbeer R, Bayerl C et al. Association of smoking and physical inactivity with MRI derived changes in cardiac function and structure in cardiovascular healthy subjects. Sci Rep 2019;9:18616. 10.1038/s41598-019-54956-8 31819090 PMC6901589

[12] Banach M, Lewek J, Surma S et al. The association between daily step count and all-cause and cardiovascular mortality: a meta-analysis. Eur J Prev Cardiol 2023;30:1975–1985. 37555441 10.1093/eurjpc/zwad229

[13] Baker LA, March DS, Wilkinson TJ et al. Clinical practice guideline exercise and lifestyle in chronic kidney disease. BMC Nephrol 2022;23:75. 10.1186/s12882-021-02618-1. 35193515 PMC8862368

[14] Tentori F, Elder SJ, Thumma J et al. Physical exercise among participants in the Dialysis Outcomes and Practice Patterns Study (DOPPS): correlates and associated outcomes. Nephrol Dial Transplant 2010;25:3050–62. 10.1093/ndt/gfq138 20392706

[15] Tudor-Locke C, Bassett DR. How many steps/day are enough? Preliminary pedometer indices for public health. Sports Med 2004;34:1–8. 10.2165/00007256-200434010-00001 14715035

[16] Tudor-Locke C, Craig CL, Thyfault JP et al. A step-defined sedentary lifestyle index: <5000 steps/day. Appl Physiol Nutr Metab 2013;38:100–14. 23438219 10.1139/apnm-2012-0235

[17] Graham-Brown MP, March DS, Young R et al. A randomized controlled trial to investigate the effects of intra-dialytic cycling on left ventricular mass. Kidney Int 2021;99:1478–86. 10.1016/j.kint.2021.02.027 34023029

[18] Graham-Brown M, March D, Churchward D et al. Design and methods of CYCLE-HD: improving cardiovascular health in patients with end stage renal disease using a structured programme of exercise: a randomised control trial. BMC Nephrol 2016;17:69. 10.1186/s12882-016-0294-7 27391774 PMC4938939

[19] American College of Sports Medicine . ACSM's Guidelines for Exercise Testing and Prescription. Baltimore: Lippincott Williams & Wilkins, 2013. 10.1249/JSR.0b013e31829a68cf 23851406

[20] Prescott S, Traynor JP, Shilliday I et al. Minimum accelerometer wear-time for reliable estimates of physical activity and sedentary behaviour of people receiving haemodialysis. BMC Nephrol 2020;21:230. 10.1186/s12882-020-01877-8 32546225 PMC7296937

[21] Hundley WG, Bluemke D, Bogaert JG et al. Society for Cardiovascular Magnetic Resonance guidelines for reporting cardiovascular magnetic resonance examinations. J Cardiovasc Magn Reson 2009;11:1. 10.1186/1532-429X-11-5 19257889 PMC2662831

[22] Graham-Brown MP, Gulsin GS, Poli F et al. Differences in native T1 and native T2 mapping between patients on hemodialysis and control subjects. Eur J Radiol 2021;140:109748. 10.1016/j.ejrad.2021.109748 33962255

[23] Graham-Brown MP, March DS, Churchward DR et al. Novel cardiac nuclear magnetic resonance method for noninvasive assessment of myocardial fibrosis in hemodialysis patients. Kidney Int 2016;90:835–44. 10.1016/j.kint.2016.07.014 27633869

[24] Lavie CJ, Ozemek C, Carbone S et al. Sedentary behavior, exercise, and cardiovascular health. Circ Res 2019;124:799–815. 10.1161/CIRCRESAHA.118.312669 30817262

[25] Durstine JL, Gordon B, Wang Z et al. Chronic disease and the link to physical activity. J Sport Health Sci 2013;2:3–11. 10.1016/j.jshs.2012.07.009

[26] Matsuzawa R, Roshanravan B, Shimoda T et al. Physical activity dose for hemodialysis patients: where to begin? Results from a prospective cohort study. J Ren Nutr 2018;28:45–53. 10.1053/j.jrn.2017.07.004 28893466 PMC6365108

[27] Ahmadi MN, Rezende LF, Ferrari G et al. Do the associations of daily steps with mortality and incident cardiovascular disease differ by sedentary time levels? A device-based cohort study. Br J Sports Med 2024;58:261–8. 10.1136/bjsports-2023-107221 38442950 PMC10958308

[28] Hardman AE, Stensel DJ. Physical Activity and Health: the Evidence Explained. London: Routledge, 2009.

[29] Wanner C, Krane V, März W et al. Atorvastatin in patients with type 2 diabetes mellitus undergoing hemodialysis. N Engl J Med 2005;353:238–48. 10.1056/NEJMoa043545 16034009

[30] Herzog CA, Strief JW, Collins AJ et al. Cause-specific mortality of dialysis patients after coronary revascularization: why don't dialysis patients have better survival after coronary intervention? Nephrol Dial Transplant 2008;23:2629–33. 10.1093/ndt/gfn038 18299298 PMC2727291

[31] Yao Q, Pecoits-Filho R, Lindholm B et al. Traditional and non-traditional risk factors as contributors to atherosclerotic cardiovascular disease in end-stage renal disease. Scand J Urol Nephrol 2004;38:405–16. 10.1080/00365590410031715 15764253

[32] Sars B, van der Sande FM, Kooman JP. Intradialytic hypotension: mechanisms and outcome. Blood Purif 2020;49:158–67. 10.1159/000503776 31851975 PMC7114908

[33] McGuire S, Horton EJ, Renshaw D et al. Cardiac stunning during haemodialysis: the therapeutic effect of intra-dialytic exercise. Clin Kidney J 2021;14:1335–44. 10.1093/ckj/sfz159 33959263 PMC8087145

[34] McGuire S, Horton EJ, Renshaw D et al. Hemodynamic instability during dialysis: the potential role of intradialytic exercise. Biomed Res Int 2018;2018:8276912. 10.1155/2018/8276912 29682559 PMC5848102

[35] Wang K, Deng Y, Xiao H. Exercise and cardiac fibrosis. Curr Opin Physiol 2023;31:100630. 10.1016/j.cophys.2022.100630

[36] Bishop NC, Burton JO, Graham-Brown MP et al. Exercise and chronic kidney disease: potential mechanisms underlying the physiological benefits. Nat Rev Nephrol 2023;19:244–56. 10.1038/s41581-022-00675-9 36650232

[37] Laverack G . The challenge of behaviour change and health promotion. Challenges 2017;8:25. 10.3390/challe8020025

[38] Li T, Lv A, Xu N et al. Barriers and facilitators to exercise in haemodialysis patients: a systematic review of qualitative studies. J Adv Nurs 2021;77:4679–92. 10.1111/jan.14960 34258784

